# Physiology of the Lymphatic System

**Published:** 1878-11

**Authors:** Z. C. McElroy


### ARTICLE IX.

### *Physiology of the Lymphatic System.*

Dr. Z. C. McElroy, (*Buffalo Med. and Surg. Journal,*) broaches a new theory with regard to the Physiology of the Lymphatic System, which is in substance that everything nature, " every special form of life, during life, or just before, or in the act of death, so to speak, makes provision for its own perpetuation and multiplication." This, he asserts, is true of animal and vegetable life, and he concludes that by some analagous process each special structure, be it viscus or tissue, in the act of functional decay, "stores up" the force in the necessary material, to carry its own and the new material, with appropriate and necessary conditions, up to the forms of structure from whence it was derived.

"The Lymph System, by this conception, becomes the 'fountain of life.' For it is the egg's seed, or germs of the several structures of the body from which it is taken, or in which it is found or derived." And, according to this theory, the Lymphatic System will have a function corresponding with its anatomical extent and complexity of structure, viz: to "collect from the debris of the tissues the material in which the several structures have stored up the force for over reproduction from new material during the life of each individual. The old is, by this means, constantly assisted with the new material steadily going into living bodies, so that personal indentity becomes intelligible. To repair the structures, as they are constantly and momentarily wasted, the supply of seed must be continuous to insure continuous reproduction." The theory has at all events the merit of novelty.

